# Insulin regulation of regional lipolysis in upper-body obese and lean humans

**DOI:** 10.1172/jci.insight.175629

**Published:** 2024-04-11

**Authors:** Søren Nielsen, Michael D. Jensen

**Affiliations:** 1 Endocrine Research Unit, Mayo Clinic, Rochester, Minnesota, USA.; 2 Steno Diabetes Center, Aarhus University Hospital, Aarhus, Denmark.; 3 Department of Clinical Medicine, Aarhus University, Aarhus, Denmark.

**Keywords:** Endocrinology, Adipose tissue, Insulin, Obesity

## Abstract

**BACKGROUND.** Upper-body obesity (UBO) results in insulin resistance with regards to free fatty acid (FFA) release; how this differs by fat depot and sex between adults with UBO and lean adults is unknown. We tested the hypothesis that insulin suppression of FFA release from the splanchnic bed, leg fat, and upper-body nonsplanchnic (UBNS) adipose tissue would be impaired in UBO.

**METHODS.** Fourteen volunteers with UBO (7 men and 7 women) and 14 healthy volunteers with normal weight (7 men and 7 women) participated in studies that included femoral artery, femoral vein, and hepatic vein catheterization. We then measured leg and splanchnic plasma flow as well as FFA kinetics (using isotopic tracers) under overnight fasting as well as low- and high-dose insulin infusion using the insulin clamp technique.

**RESULTS.** We found the expected insulin resistance in UBO; the most quantitatively important difference between adults with UBO and lean adults was greater FFA release from UBNS adipose tissue when plasma insulin concentrations were in the postprandial, physiological range. There were obesity, but not sex, differences in the regulation of splanchnic FFA release and sex differences in the regulation of leg FFA release.

**CONCLUSION.** Reversing the defects in insulin-regulated UBNS adipose tissue FFA release would have the greatest effect on systemic FFA abnormalities in UBO.

**FUNDING.** These studies were supported by the US Public Health Service (grants DK45343 and DK40484), the Novo Nordic Foundation (grant NNF18OC0031804 and NNF16OC0021406), and the Independent Research Fund Denmark (grant 8020-00420B).

## Abstract 2

Insulin-regulated regional free fatty acid metabolism is impaired in upper-body obesity during moderate hyperinsulinemia, especially in upper-body subcutaneous adipose tissue.

## Graphical abstract

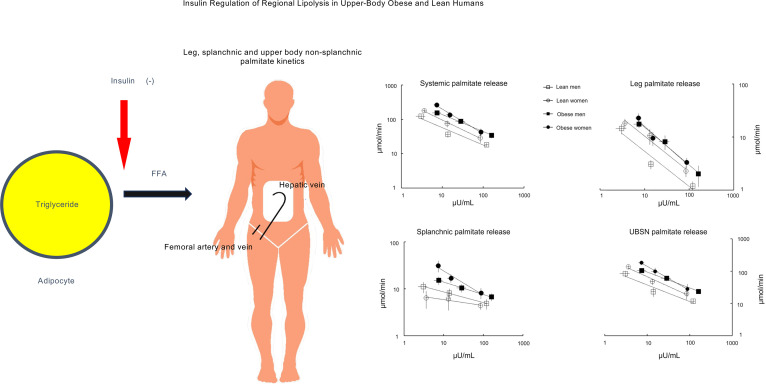

## Introduction

Upper-body obesity (UBO), especially visceral obesity, is an insulin-resistant condition characterized by increased prevalence of glucose and lipid metabolic abnormalities, hypertension, and cardiovascular disease (1, 2). The metabolic abnormalities include impaired glucose tolerance, type 2 diabetes, dyslipidemia, and increased plasma free fatty acid (FFA) concentrations (2). Specifically, and impaired ability of insulin to suppress adipose tissue lipolysis (3) and hepatic glucose and triglyceride secretion (4) and to promote glucose disposal in peripheral tissues has been well described. The increased FFA flux is likely causally involved in these processes since many of the metabolic complications can be reproduced in lean individuals by increasing plasma FFA experimentally. Increased hepatic FFA delivery augments hepatic gluconeogenesis and triglyceride production (5, 6). In addition, we previously reported that the fraction of postabsorptive hepatic FFA delivery originating from visceral adipose tissue lipolysis increases proportionally with size of the visceral fat depot and, in some individuals, could account for more than 40% of hepatic FFA exposure (7).

In adults with or without obesity, the vast majority of the postabsorptive systemic FFA flux originates from upper-body nonsplanchnic (UBNS) (i.e., subcutaneous) lipolysis (7, 8). Moreover, hepatic FFA delivery is less suppressed after a mixed meal in women with UBO compared with women with lower-body obesity (9). In lean individuals, the relative contribution of visceral fat lipolysis to total systemic FFA turnover increases proportionately with increments in experimental hyperinsulinemia (8). This relationship could be further augmented in visceral obesity. Lipolysis from visceral fat adipocytes in vitro is less suppressible by insulin than that from adipocytes from UBNS fat and leg fat (10). Thus, if visceral fat is more resistant to the antilipolytic effect of insulin in vivo, visceral adipose tissue lipolysis could contribute directly to the metabolic abnormalities of visceral/UBO.

It is currently unknown whether the sensitivity of regional adipose tissue lipolysis (subcutaneous vs. visceral) to insulin differs in vivo between men and women who are lean versus those with UBO. Moreover, it is unknown whether hyperinsulinemia per se alters the relative contribution of regional lipolysis to systemic FFA flux and the proportion of hepatic FFA delivery coming from the visceral adipose tissue. Such data could reveal sex- and obesity-specific differences of the quantitative degree of hepatic FFA exposure during hyperinsulinemia and may help design depot-specific strategies for improved postprandial suppression of regional lipolysis.

The purpose of these studies was to examine whether the insulin sensitivity with respect to antilipolysis of visceral and subcutaneous adipose tissue differs between individuals with UBO and lean individuals and whether such differences alter the contribution of visceral adipose tissue lipolysis to hepatic FFA delivery and systemic FFA flux. In addition, we included equal numbers of male and female participants so we would be able to explore whether there are sex-specific differences in regional FFA metabolism. To accomplish this, we measured systemic FFA flux in combination with regional (splanchnic, UBNS, and leg) FFA balances in men and women who were either normal weight (BMI, 18–25 kg/m^2^) or had UBO based on anthropometric criteria. Measurements were performed in the basal state and during a 2-step hyperinsulinemic, euglycemic clamp using palmitate tracer technique in combination with catheterization of the hepatic vein and the femoral artery and vein. We hypothesized that the relative contribution of visceral fat lipolysis to both systemic lipolysis and to hepatic fat delivery increases proportionally with insulin concentration and that the proportions will be greater in adults with UBO than lean adults.

## Results

### Participant characteristics.

As expected, visceral and abdominal subcutaneous fat area, total and percentage body fat, and fat-free mass (FFM) were all greater in volunteers with a UBO phenotype compared with their normal weight counterparts (Table 1). Resting energy expenditure (REE) and respiratory exchange ratio (RER) data for the basal, low insulin dose, and high insulin dose clamp intervals are provided in Table 2. For the combined groups (male vs. female, obese vs. lean), REE was greater in male participants than in female participants (*P* < 0.05), but RER values under the basal and low- and high-dose insulin infusions were not different between sexes. REE was greater in volunteers with obesity versus those with normal weight (*P* < 0.01), and RER was significantly lower in those with obesity during all 3 intervals (at least *P* < 0.01).

There were no statistical differences between the 4 groups in splanchnic or leg plasma flow during the basal or low insulin or high insulin dose intervals (1-way ANOVA). In lean men only, splanchnic plasma flow increased significantly from the basal and low insulin dose intervals to the high-dose interval (*P* < 0.001, Table 2). Leg plasma flow rates after basal, low insulin dose, and high insulin dose insulin infusions did not change significantly in any of the groups. Because the indocyanine green (ICG) infusion was not started properly during the low-dose insulin infusion for 1 man with obesity, we imputed leg and splanchnic plasma flow for that interval using data from the preceding and following intervals.

### Systemic palmitate, insulin, and catecholamine concentrations.

**Figure 1:**
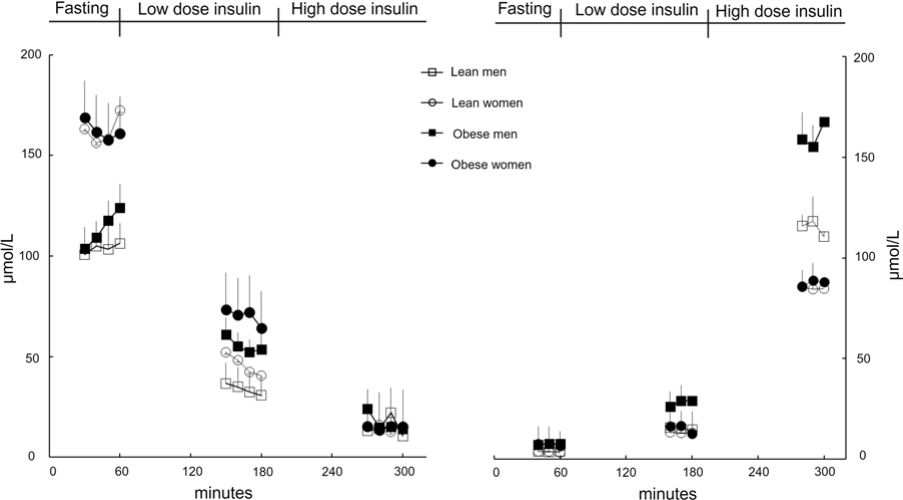
Palmitate and insulin concentrations. Plasma palmitate concentrations (left) and insulin concentrations (right) under overnight postabsorptive conditions (time points, 30–60 min), low-dose insulin infusion (150–180 min), and high-dose insulin infusion (270–300 min) are plotted for 7 men and 7 women in the lean group and 7 men and 7 women in the upper-body obesity group. Values are shown as mean ± SEM.

Figure 1 shows plasma palmitate and insulin concentrations during the basal and low- and high-dose insulin infusions. The stepwise increases in insulin concentrations resulted in stepwise suppression of palmitate concentrations. Plasma catecholamine concentrations for the entire group are provided in Supplemental Figure 1 (supplemental material available online with this article; https://doi.org/10.1172/jci.insight.175629DS1); norepinephrine increased significantly from the basal period (120 ± 50 nmol/L) to the first insulin dose (153 ± 56 nmol/L, *P* < 0.0001) to the second insulin dose (171 ± 55 nmol/L, *P* = 0.04 vs. first dose, *P* < 0.0001 vs. basal); there were no statistically significant between-group differences. Likewise, for the entire group, plasma epinephrine concentrations increased significantly from the basal period (30 ± 19 pmol/L) to the first insulin dose (46 ± 36 pmol/L, *P* = 0.014) to the second insulin dose (62 ± 55 pmol/L, *P* = 0.016 vs. first dose, *P* = 0.003 vs. basal), and there were no statistically significant between-group differences. There was substantial interindividual heterogeneity in the catecholamine changes between the 3 study intervals; intraindividual differences in plasma epinephrine concentrations ranged from as little as 0 nmol/L to as much as 155 pmol/L, and intraindividual differences in plasma norepinephrine concentrations ranged from as little as 20 nmol/L to as much as 205 nmol/L.

### Systemic palmitate kinetics.

**Figure 2:**
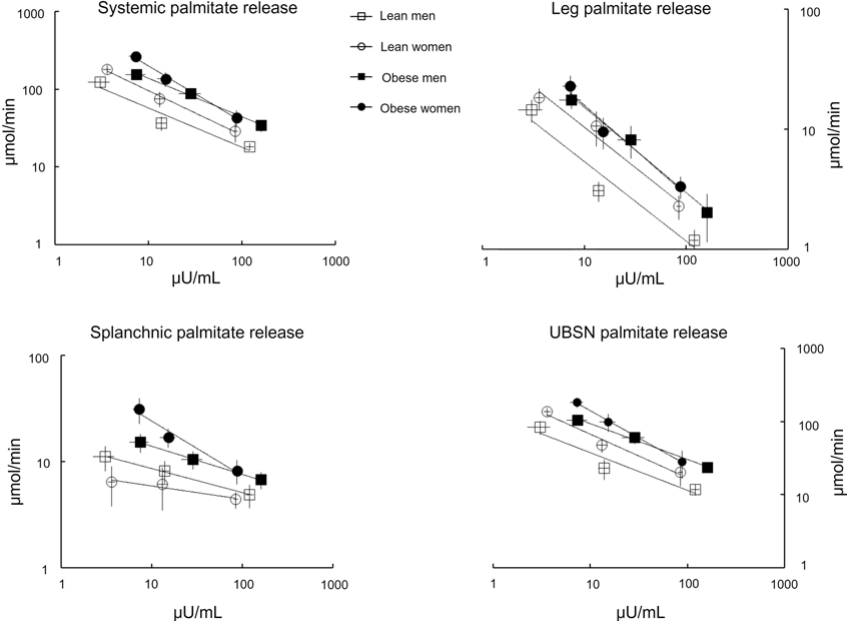
Systemic and regional palmitate release rates. Palmitate rates of appearance are plotted versus measured plasma insulin concentrations for 7 men and 7 women in the lean group and 7 men and 7 women in the upper-body obesity group. The relationships for systemic palmitate rates of appearance and insulin concentrations are depicted in the top left, for leg palmitate rate of appearance in the top right, for splanchnic palmitate rate of appearance in the bottom left, and upper-body nonsplanchnic (UBNS) in the bottom right. Values are shown as mean ± SEM.

Basal systemic palmitate rate of appearance was different between groups (*P* = 0.001, ANOVA) and was significantly greater in women with the UBO phenotype compared with that in the other 3 groups (*P* < 0.02). The mean ± SEM palmitate rate of appearance values are provided in Table 3 and depicted in Figure 2. The individual systemic palmitate rate of appearance values for female (Supplemental Figure 2) and male participants (Supplemental Figure 2) are also provided.

For the entire group, palmitate rate of appearance was sequentially suppressed at each insulin dose (all *P* < 0.001; Figure 2), though there were two exceptions. For 1 lean female participant, plasma insulin concentrations increased by only 6 μIU/mL in response to the first insulin dose, and her plasma epinephrine concentrations increased by 76 pmol/L; her palmitate release rates did not decrease during that clamp interval. The individual data showing this (and all) response are provided in Supplemental Figure 2. We did not calculate the systemic or regional IC_50_ values for this volunteer because the increase in her plasma epinephrine concentrations was well into range that stimulates lipolysis (11). For 1 lean male participant, we achieved maximal suppression of palmitate release at the first insulin dose (increase in plasma insulin concentrations of 14 μIU/mL), as evidenced by failure of palmitate release to decrease in response to the 108 μIU/mL increase in plasma insulin concentrations during the second insulin dose (demonstrated in Supplemental Figure 2, top right); the IC_50_ for this volunteer was calculated using basal and low insulin dose data. The IC_50_ could not be calculated for the lean male volunteer who did not receive the palmitate tracer infusion during the basal interval. The IC_50_ for systemic palmitate release for each group is provided in Table 3. As expected, the IC_50_ values were significantly greater in the obese groups than lean groups.

### Leg palmitate release.

Basal leg palmitate release rates were not significantly different between groups, whether expressed as absolute amounts or as a percentage of systemic palmitate flux (Table 3 and Figure 2).

Leg palmitate release (μmol/min) was sequentially suppressed by low and high insulin doses in both obese (*P* at least < 0.001) and lean (*P* at least < 0.01) volunteers (Figure 2, top right for mean data, and Supplemental Figure 2, bottom for individual data). When comparing leg palmitate release, we found no significant differences, either as absolute amounts or as percentage of systemic release, between obese and lean volunteers under basal or low- or high-dose insulin conditions. We could calculate the leg palmitate release IC_50_ for 12 lean volunteers and 14 volunteers with obesity; the values for obese and lean participants were 25 ± 9 μIU/mL and 7 ± 1 μIU/mL, respectively (*P* = 0.07).

**Figure 3:**
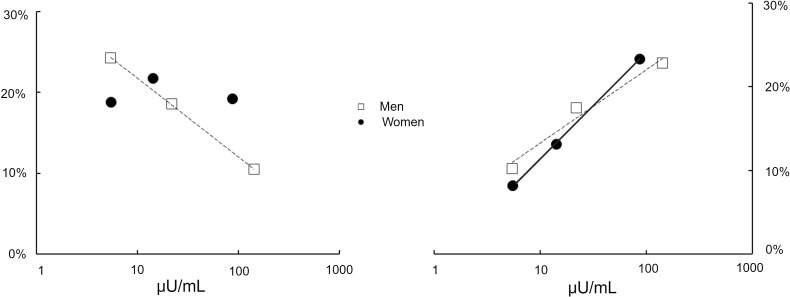
Leg and splanchnic palmitate release in response to insulin. The mean percentage contribution of leg palmitate release to total palmitate release is plotted versus mean plasma insulin concentrations for men and women (left). There are 7 men and 7 women in the lean group and 7 men and 7 women in the upper-body obesity group. Correlations were evaluated by Pearson’s *r*. There was a negative relationship (*r* = –0.99) for men but not for women. The mean percentage contribution of splanchnic palmitate release to total palmitate release is plotted versus mean plasma insulin concentrations for men and women (right). There was a positive relationship (*r* = 0.97) for women and for men (*r* = 0.88).

We also compared the leg palmitate release responses of male and female participants independent of obesity. The contribution of basal leg palmitate release to total palmitate release was not different between male and female participants. During the high insulin dose, the percentage of systemic palmitate release from leg was greater in female than male participants (20% ± 2% vs. 11% ± 2%, respectively, *P* = 0.04), but the difference in absolute leg palmitate release rates (2.8 ± 0.3 μmol/min vs. 1.6 ± 0.4 μmol/min, respectively) did not reach statistical significance (*P* = 0.06). Of interest, there was a negative correlation between mean percentage contribution of leg palmitate release to total palmitate release and mean log-transformed plasma insulin concentrations in male but not female participants (Figure 3).

### Splanchnic palmitate release.

Basal splanchnic palmitate release rates are depicted in Figure 2 and provided in Table 3. By ANOVA there were between-group differences in absolute splanchnic palmitate release rates under basal (*P* = 0.01) and low insulin dose (*P* = 0.04) conditions but not under high insulin dose conditions (*P* = 0.26). When comparing the participants with obesity to the lean participants without regards to sex, splanchnic palmitate release was greater in the participants with obesity under basal (23 ± 5 μmol/min vs. 9 ± 1 μmol/min, respectively, *P* = 0.01), low-dose insulin (14 ± 2 μmol/min vs. 7 ± 1 μmol/min, respectively, *P* = 0.02), and high-dose (8 ± 1 μmol/min vs. 5 ± 1 μmol/min, respectively, *P* = 0.049) conditions. In contrast, splanchnic palmitate release as a percentage of total palmitate release was not statistically different between the obese and lean groups under any of the 3 conditions. The percentage of systemic palmitate coming from splanchnic palmitate release increased with each increment in insulin concentrations in the lean volunteers (all *P* < 0.02), but the increase was only statistically significant with the high insulin dose for the volunteers with obesity (*P* < 0.05 vs. both baseline and low-dose insulin). Due to the large variability in the responses of splanchnic palmitate release to low and high insulin conditions we were unable to calculate IC_50_ values for this tissue bed.

We also compared the responses of male and female participants irrespective of obesity. There were no statistically significant differences in splanchnic palmitate release, either in absolute amounts or as a percentage (Figure 3) of total palmitate release between male and female participants.

### UBNS palmitate release.

UBNS palmitate release rates are provided in Table 3 and Figure 2. The individual UBNS palmitate rate of appearance values for female (Supplemental Figure 2, middle left) and male (Supplemental Figure 2, middle right) participants are also provided. Basal UBNS palmitate release was significantly different between groups (*P* < 0.005, ANOVA) due to a lesser release rate in lean men compared with the other 3 groups (*P* < 0.05). Hyperinsulinemia resulted in a significant suppression of UBNS palmitate release in all groups (all *P* < 0.001).

When comparing the participants with UBO to the lean participants, basal UBNS palmitate release was not statistically different whether expressed as absolute values (144 ± 18 μmol/min vs. 105 ± 10 μmol/min, respectively, *P* = 0.11) or as a percentage of systemic palmitate release (68% ± 3% vs. 71% ± 3%, respectively, *P* = NS). However, under conditions of low-dose insulin, UBNS palmitate release was greater in the UBO group than in the lean group in absolute amounts (80 ± 15 μmol/min vs. 36 ± 5 μmol/min, respectively, *P* = 0.02) but not as a percentage of systemic release (67% ± 5% vs. 61% ± 3%, respectively, *P* = NS). Under conditions of high-dose insulin, UBNS palmitate release was not statistically different in the UBO and lean groups in either absolute amounts (26 ± 6 μmol/min vs. 16 ± 2 μmol/min, respectively) or as a percentage of systemic release (59% ± 8% vs. 63% ± 3%, respectively). UBNS palmitate release rates (μmol/min) were significantly (at least *P* < 0.001) sequentially suppressed by low- and high-dose insulin in both volunteers with obesity and lean volunteers. The percentage of palmitate release contributed by UBNS adipose was not different among basal or low and high insulin dose conditions in the lean and obese groups. The estimated IC_50_ values for UBNS palmitate release in obese (*n* = 14) and lean (*n* = 12) volunteers were 25 ± 15 μIU/mL and 9 ± 2 μIU/mL, respectively (*P* = 0.002).

We also compared the responses of male and female participants irrespective of obesity. Under basal conditions UBNS palmitate release was greater in female participants than in male participants (161 ± 11 μmol/min vs. 95 ± 11 μmol/min, respectively, *P* = 0.003), but there were no significant sex differences under the low- or high-dose insulin conditions. There were no sex differences in the percentage of systemic palmitate release coming from UBNS adipose tissue.

The IC_50_ values for UBNS adipose tissue palmitate release for participants who were lean and had UBO were 9.1 ± 1.6 (*n* = 12) and 27.2 ± 4.2 (*n* = 13) μIU/mL, respectively (*P* = 0.0003).

### Regional palmitate uptake.

Leg and splanchnic palmitate uptake rates are provided in Table 3; for all groups combined, both were significantly, sequentially suppressed by each insulin dose. When comparing UBO and lean groups, the only significant difference was greater splanchnic palmitate uptake under low-dose insulin conditions in the obese group (24 ± 3 vs. 14 ± 1 μmol/min, respectively, *P* = 0.03). If the data from the lean female participant whose palmitate concentrations were not suppressed during the low-dose insulin interval (presumably related to the exaggerated epinephrine response) were included, leg palmitate uptake under low-dose insulin conditions was greater in female participants than in male (8 ± 1 μmol/min vs. 5 ± 1 μmol/min, respectively, *P* = 0.046); the difference was not significant (*P* = 0.09) absent that data point.

### Splanchnic and hepatic palmitate delivery.

Splanchnic plasma flow was not significantly different among the basal or low-dose and high-dose insulin clamp intervals, thus the suppression of splanchnic palmitate delivery (Table 4) in response to insulin was the result of reduced plasma palmitate concentrations. Splanchnic palmitate delivery was greater in participants with obesity than participants without obesity only during the low-dose insulin clamp (*P* = 0.003).

**Figure 4:**
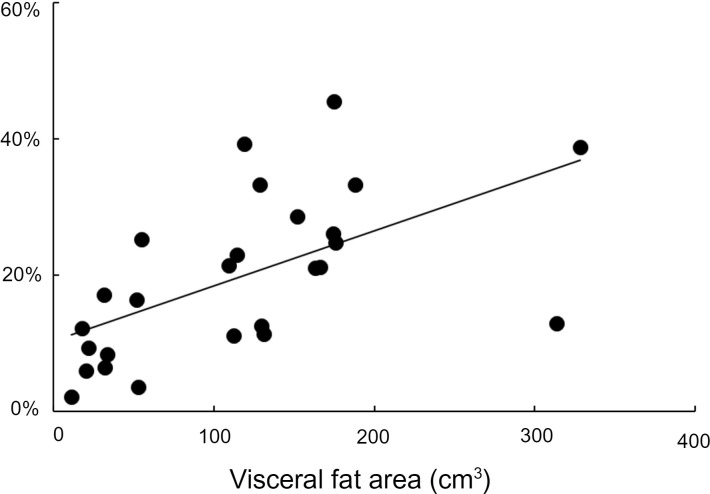
Proportion of hepatic palmitate delivery from visceral fat under baseline conditions. The relationship between visceral fat area as measured by abdominal CT versus the percentage of hepatic palmitate delivery originating from visceral adipose tissue under basal conditions (*r* = 0.58, *P* < 0.005) is depicted. There are 7 men and 7 women in the lean group and 7 men and 7 women in the upper-body obesity group. Correlations were evaluated by Pearson’s *r*. Mean values are depicted.

The proportion of basal hepatic palmitate delivery originating from visceral adipose tissue palmitate release was significantly greater in the obese than nonobese groups (26% ± 3% vs. 12% ± 2%, *P* = 0.001). Although there was not a significant relationship between basal splanchnic palmitate release and visceral fat area (*r* = 0.19, *P* = 0.34), there was a positive relationship between visceral fat area as measured by abdominal CT and the percentage of hepatic palmitate delivery originating from visceral adipose tissue under basal conditions (*r* = 0.58, *P* < 0.005, Figure 4). The percentage of hepatic palmitate delivery originating from visceral adipose tissue increased during each dose of insulin (Table 4) and was not different between obese and nonobese groups during low and high insulin clamp intervals. There was no relationship between visceral fat area by CT and the percentage of hepatic palmitate delivery originating from visceral adipose tissue under either of the hyperinsulinemic conditions.

## Discussion

We have previously examined the regulation of regional fatty acid metabolism by insulin alone in nonobese volunteers (8) and in adults with obesity and type 2 diabetes versus obesity alone (12). We have also investigated the effect of meal ingestion on regional fatty acid metabolism in adults with obesity (9) and those who are nonobese (13). However, our review of the literature indicates that how insulin alone regulates regional FFA metabolism in adults who are nonobese and obese has not been studied. We investigated the differences in regional FFA kinetic responses to both modest and maximally effective insulin concentrations in adult men and women with a normal BMI and in those with UBO because of the known sex (14–16) and adiposity (17) differences in adipose tissue metabolism. We found that leg adipose lipolysis varied both as a factor of obesity and sex, in that the IC_50_ for suppression of FFA release was greater in adults with obesity than in nonobese adults, and the fractional contribution of leg adipose FFA release decreased in men, but not women, in response to progressively increased plasma insulin concentrations. There were obesity, but not sex, differences in the insulin regulation of splanchnic FFA release as well as evidence of both obesity and sex effects in the insulin regulation of UBNS lipolysis. In summary, these studies show that both sex and obesity influence insulin regulation of adipose tissue lipolysis in a depot-specific fashion.

Our volunteers with obesity were screened to increase the likelihood they would have the typical adipose tissue insulin resistance with regards to lipolysis that we have come to expect (18) in those with class I obesity. Indeed, obesity in our volunteers was accompanied by greater amounts of visceral fat; reduced metabolic flexibility, as measured by the ability of insulin to increase RER; and by a 2- to 3-fold greater IC_50_ for suppression of adipose tissue palmitate release. The reduced ability of insulin to suppress lipolysis in obesity was most evident in response to the low-dose insulin infusion (Figure 1 and Table 3). During the high-dose insulin infusion that achieved plasma insulin concentrations greater than those seen postprandially, differences in FFA release rates were less evident. Thus, with sufficient insulin stimulation it is possible for adipocytes in UBO to suppress lipolysis to nearly the same extent as in nonobese adults. These findings suggest that the optimal range of insulin concentrations to study defects in UBO adipose responsiveness are closer to those observed after meal ingestion, which in any case is most physiologically relevant. We note that the dose of insulin we selected to achieve maximally suppressive effects on FFA release created sufficiently high plasma insulin concentrations that we likely underestimated the insulin sensitivity in our lean control group.

The greater systemic palmitate IC_50_ values in volunteers who had UBO compared with lean volunteers were, in a quantitative sense, mostly due to differences in UBNS palmitate release, although insulin resistance was present in leg fat of volunteers with UBO. We found that UBNS adipose tissue accounted for well over 60% of systemic FFA release in all groups under most conditions, reemphasizing the fact that this tissue bed is of central importance in the excess FFA availability that is a feature of UBO. For example, palmitate release from UBNS fat under low-dose insulin conditions was 2–3 times (40 – 50 μmol/min) greater in volunteers who were UBO than those who were lean, whereas under the same conditions average leg (0–10 μmol/min) and splanchnic (also 0–10 μmol/min) palmitate release was marginally greater in those who had UBO. Analysis of adipocyte protein responses to insulin in this more readily accessible abdominal subcutaneous fat could help dissect the mechanisms of systemic insulin resistance in UBO for both male and female participants. Given the apparent sex differences in the relative effects of insulin on leg adipose tissue FFA release (Figure 3, left), the study of adipocyte lipolysis pathways using biopsy specimens from leg fat should provide greater insights into systemic insulin resistance for female compared with male participants. The potential mechanisms for adipose tissue insulin resistance of UBO range from reduced delivery of insulin to adipose tissue (19), to impaired insulin signaling (20), to a selective reduction in the responsiveness of lipolysis-regulatory proteins downstream of the canonical insulin signaling pathway (21, 22). The latter two possibilities can be interrogated using carefully timed biopsy specimens.

However, interpreting the results from adipose samples in the context of insulin resistance with respect to lipolysis will be somewhat challenging. For example, how much of the excess FFA release from UBNS adipose tissue is due to a fundamental dysfunction at the tissue level as opposed to reflecting the excess adipose tissue mass in obesity? By measuring regional lipolysis, we could calculate that, during the low-dose insulin infusion, the mean palmitate release rates from UBNS fat were 4.5, 3.0, 4.4, and 4.7 μmol•kg/fat/min in lean women, lean men, women with UBO, and men with UBO, respectively. One interpretation is that the greater mass of UBNS adipose tissue in the UBO groups (approximately double that in the lean groups) is responsible for the excess palmitate release, rather than any inherent defect in lipolysis regulation at the tissue level. However, when we studied women who are equally obese with a lower-body fat distribution, we found that total (9, 17, 23, 24) and regional (9, 23, 24) FFA release rates per kg fat were reduced compared with volunteers who were lean and UBO, resulting in systemic FFA release rates that are much closer to those observed in nonobese women. This suggests that fat gain in those with a lower-body obesity phenotype occurs in an adaptive manner to maintain a normal FFA environment. To better understand this phenomenon, including participants with a lower-body obesity phenotype as well as nonobese participants with a predominant upper-body fat distribution in future studies of regional FFA kinetics and adipocyte responsiveness (using adipose biopsies) will be helpful.

Unfortunately, it is not possible to study the insulin responsiveness of visceral adipose tissue using biopsy/in vivo functional approaches in humans. In this study, and consistent with our previous observations (7), the fractional delivery of FFA to the liver from visceral adipose tissue was positively correlated with visceral fat. Understanding the regulation of FFA release from omental and mesenteric adipocytes in the context of these observations will require the study of adipose tissue collected from carefully selected surgical patients rather than research volunteers. By collecting hepatic vein blood samples while measuring splanchnic plasma flow and infusing a palmitate tracer, we were able to show that baseline splanchnic palmitate release originating from visceral fat was greater in those with obesity. However, splanchnic palmitate release rates, in contrast to the fractional delivery of FFA to the liver from visceral adipose tissue, were not correlated with visceral adipose area measured by CT. The most logical explanation for these seemingly contradictory findings is that the clearance of portally delivered FFA by the liver varies considerably between individuals. We previously reported that there are errors in calculating the fractional delivery of FFA to the liver from visceral fat, especially under conditions of low FFA concentrations (25), such as were observed in these studies. However, these errors seem unlikely to account for the lack of relationship between visceral fat and the fractional delivery of FFA to the liver from visceral fat under hyperinsulinemic conditions; these estimates were very similar in all groups (Table 4). To the extent that our findings represent the potential for excess hepatic FFA delivery to be important, the biggest relative difference between the lean and obese states under low insulin conditions seems to be due to the greater splanchnic delivery of FFA from the systemic circulation.

As with all studies, we acknowledge that ours has limitations. Due to the intensive nature of the experiments, we had relatively small numbers of participants in each group. This prevented us from assessing whether the differences in body fat distribution in adults with normal weight is related to adipose insulin resistance. In addition, these studies were designed to detect the anticipated effects of UBO on adipose tissue FFA release but not sex-specific effects. Thus, the lack of differences between male and female participants in some of the responses cannot be taken as proof of sameness. However, the large differences between obese and lean participants in terms of adipose tissue insulin action allowed us to detect highly significant differences. We used a palmitate tracer to study FFA release; however, palmitate represents only approximately 25% of plasma FFA. Investigators who have studied humans using multiple FFA tracers have concluded that palmitate is a reasonable choice to represent FFA release rates under a variety of conditions (26). Another consideration is that the invasive nature of the study (femoral artery and vein catheters) could have affected the responses to the insulin infusion; as evidenced by the variable increases in plasma catecholamine concentrations we observed. These adrenomedullary and sympathetic nervous system activation responses may have partially offset the antilipolytic effects of insulin (11), and we may have underestimated the potency of insulin per se (compared with isolated adipocytes). The finding that catecholamines increased with each insulin dose is consistent with previous observations (27) and may indicate that this response is an inherent, in vivo effect of hyperinsulinemia in humans and, thus, not an aberrant feature of our study. However, the strengths of our study include the use of validated tracer techniques, measurement of plasma flow with the accepted (ICG) approach, and careful prestudy control over diet.

In summary, the most quantitatively significant abnormality of lipolysis regulation by insulin in UBO is in the greater FFA release by the UBNS depot under moderately increased insulin concentrations. The ability of insulin to regulate lipolysis, as reflected by a greater IC_50_ for suppression of palmitate release, was impaired in both leg and UBNS fat in those with obesity. We believe that this is important, because attention to the regulation of FFA release from the UBNS depot can help us understand the lipotoxicity of UBO. Of note, although visceral fat is associated with greater hepatic FFA delivery from visceral adipose tissue lipolysis in the fasting state, we found that the systemic circulation is the primary contributor to elevated hepatic FFA delivery under moderate hyperinsulinemic conditions; the delivery of FFA to the splanchnic bed was twice as great in participants with obesity as in participants without obesity in this study. Future studies of regional lipolysis should benefit from the inclusion of humans with lower-body obesity and should include sampling of subcutaneous fat to test the regulatory pathways that contribute to adipose insulin resistance.

## Methods

### Sex as a biological variable.

Sex as a biological variable was addressed by including equal numbers of male and female participants in these studies.

### Participants.

Fourteen, self- and investigator-identified White volunteers (7 men and 7 women) with UBO, aged 22–50 years (BMI, 28–36 kg/m^2^) and 14 healthy, normal weight volunteers (7 men and 7 women, BMI, <25 kg/m^2^), matched for age, participated in these studies. UBO was defined as a waist-to-hip of more than 0.95 in men and more than 0.85 in women. All participants had been weight stable for the last 3 months prior to the study. Weight maintaining meals (40% fat, 40% carbohydrate, 20% protein) were provided at the Mayo Clinic General Clinic Research Center (GCRC) for 3 days before the study to assure consistent macronutrient and energy intake. Volunteers were asked to maintain their usual level of physical activity and not to participate in heavy exercise for 3 days before the study. All volunteers were nonsmokers and used no regular medications. Women were premenopausal and had a negative pregnancy test prior to the study.

### Materials.

[9,10-^3^H]palmitate was purchased from Amersham Life Sciences Inc., and ICG (Cardio-Green) was purchased from Becton Dickinson.

### Protocol.

One week before the study total body fat and FFM were measured by dual-energy x-ray absorptiometry (DXA) (Lunar Radiation Corp.). Intra-abdominal and abdominal subcutaneous fat mass was assessed using a single-slice CT scanning of the abdomen at the L_2–3_ interspace in combination with the abdominal fat mass measured by DXA (28). Each participant was admitted to the GCRC the evening before the study. An 18-gauge intravenous catheter was inserted in a forearm vein and kept open with 0.45% saline. Each participant received a 325 mg aspirin tablet with the evening meal the day before the study to reduce the possibility of developing catheter-related platelet microthrombi. Studies were performed in the GCRC after a 12-hour fast. Indirect calorimetry was performed in the morning before the participants underwent other study procedures.

After the indirect calorimetry participants were transferred to the Department of Vascular Radiology for placement of catheters under sterile conditions into the hepatic vein, right femoral artery, and right femoral vein. A 5 French sheath was introduced into the right femoral artery using a standard percutaneous technique. A 20 cm–long 4 French straight catheter with 6 distally placed side holes was placed through the sheath with the catheter tip in the common iliac artery. This catheter was used for arterial blood sampling, and the sheath was used to infuse ICG. The right femoral vein was then punctured in a similar manner, and a 6 French sheath was introduced. The distal tip of the sheath was placed in the external iliac vein a few centimeters above the inguinal ligament. A 5 French Simmons 2 catheter with 4 distal side holes was advanced through the sheath, and the catheter tip was placed in the right hepatic vein. A solution of 0.45% NaCl was infused through the sheaths and catheters to maintain patency.

The volunteers were then transferred back to the GCRC for completion of the study. Blood was sampled before starting the isotope and the ICG infusions to be used for background palmitate-specific activity (SA) and for construction of the ICG calibration curve. Infusions of [9,10-^3^H]palmitate and ICG were begun 30 minutes before blood sampling. The isotopes were infused through the forearm vein catheter, and the ICG was infused into the femoral artery sheath to allow measurement of leg and splanchnic plasma flow. Arterial, femoral venous, and hepatic venous blood samples were taken at 10-minute intervals over a period of 30 minutes. Between sampling all catheters were kept patent by infusion of 0.45% saline. The palmitate tracer was accidentally not infused for 1 lean male participant during the baseline interval but was infused during the first and second insulin doses. After completion of the study, all catheters were removed, and local hemostasis was obtained. The participants remained in the hospital under observation until the following morning.

### Palmitate kinetics.

A 2-step hyperinsulinemic, euglycemic glucose clamp in combination with indirect calorimetry and regional plasma flow measurements were used to assess insulin-regulated systemic and regional palmitate kinetics (Figure 1). Insulin (Humulin insulin, Lilly) was infused at rates of 0.25 (step 1) and 2.5 mU•kg/FFM/min (step 2). Plasma glucose was measured in duplicate every 5–10 minutes immediately after sampling (Beckman Instruments), and plasma glucose concentrations were clamped at approximately 5 mmol/L during both steps by a variable infusion of 50% dextrose. Blood samples for determination of plasma palmitate concentrations and SA, ICG, insulin, growth hormone,r and catecholamine concentrations were drawn at 10-minute intervals during the last 30 minutes of the basal and hyperinsulinemic intervals.

Basal and insulin suppressed systemic palmitate flux and regional balances were measured at the end of the basal and each of the hyperinsulinemic steps using isotope dilution technique in combination with arteriovenous blood sampling. We infused [9,10-^3^H]palmitate (0.3 μCi/min) during the last hour of each interval to assure isotopic steady state before blood sampling began. The infusion pump was turned off at the end of each period.

### Indirect calorimetry.

REE and RERs were measured by indirect calorimetry (DeltaTrac Metabolic Cart; SensorMedics Corp.) with a ventilated hood during the last 30 minutes of the basal and hyperinsulinemic periods. The equipment was calibrated against standard oxygen and CO_2_ gases before each measurement.

### Plasma flow.

ICG was prepared for infusion as 75 mg of ICG in 30 mL diluent. The ICG was infused at a rate of 250 μg/min into the femoral artery sheath, allowing us to measure both leg and splanchnic plasma flow. As outlined above, arterial, femoral venous, and hepatic venous blood samples were taken at 10-minute intervals over a period of 30 minutes at the end of the basal and each of the hyperinsulinemic steps. The ICG infusion was begun 30 minutes before each sampling period and turned off at the end of each period. Blood samples were analyzed using spectrophotometry on the day of the study.

### Analysis of samples.

Plasma palmitate concentration and SA were determined by HPLC (29) using [^2^H_31_]palmitate as an internal standard (30). We have previously confirmed that our sample preparation does not result in vitro hydrolysis of triglycerides fatty acids or phospholipids.

The isotopic purity of the tracer was determined using HPLC. Plasma glucose concentrations were measured using the glucose oxidase method. Plasma concentrations of catecholamines were measured using HPLC (31), and insulin was measured using a chemiluminescent sandwich assay (Sanofi Diagnostics Pasteur Inc.). Plasma total cholesterol, HDL-cholesterol, and triglycerides were measured by enzymatic methods.

### Calculations.

Splanchnic plasma flow was calculated by dividing the ICG infusion rate by the arterial-hepatic venous concentration gradient of the dye. Leg plasma flow was calculated by dividing the dye infusion rate by the arterial-venous gradient across the leg. Systemic palmitate flux was determined using the isotope dilution technique and steady-state equations. Regional palmitate kinetics across the splanchnic and leg tissues were calculated as described previously (7). In brief, systemic palmitate flux was calculated using the average arterial palmitate SA divided by the tracer infusion rate corrected for isotopic purity. Steady-state plasma palmitate concentrations and SA were used together with leg and splanchnic plasma flow to measure regional palmitate uptake and release. UBNS palmitate release was calculated as follows: total palmitate release – [(leg palmitate release ×2) + splanchnic palmitate release]. The proportionate contribution of visceral lipolysis to hepatic FFA delivery was calculated using the hepatic vein and femoral artery palmitate SA values as previously described (7, 25).

The insulin concentration calculated to result in a 50% suppression of palmitate release (IC_50_) was calculated using each individual’s log-transformed baseline, low-dose, and high-dose plasma insulin concentrations and palmitate release rates as previously described (18); we calculated the IC_50_ values for systemic palmitate release as well as leg and UBSN depot palmitate release.

### Statistics.

Values are shown as the mean ± SEM or median (range) except where indicated. Baseline comparisons between groups were done using 2-tailed Student’s *t* test, Mann-Whitney 2-sample test, or 1-way ANOVA followed by pairwise comparisons in cases of statistical significance. The insulin dose-response effect on palmitate kinetics was analyzed using a linear regression model, and the insulin concentration resulting in a 50% suppression was calculated as previously described (18). Correlations were evaluated by Pearson’s *r* or Spearman’s *rho*. *P* values of less than 0.05 were considered statistically significant.

The statistical power for these studies was based upon previous data indicating that palmitate release rates during hyperinsulinemia would be approximately double (97 ± 10 vs. 53 ± 5 micromole/minute) in adults with UBO compared with lean controls (23). We were unable to perform power calculations regarding the potential differences in IC_50_ at the time the studies were designed.

### Study approval.

This study was approved by the IRB at Mayo Clinic, and written informed consent was obtained from all participants prior to the studies. At the time this study was approved it was not considered a clinical trial.

### Data availability.

Data are available in the supplemental materials. Values for all data points in graphs are reported in the Supporting Data Values file.

## Author contributions

SN conducted the experiments, acquired the data, analyzed the data, and wrote the manuscript. MDJ designed the research studies, acquired the data, analyzed the data, provided reagents, and wrote the manuscript.

## Supplementary Material

Supplemental data

Supporting data values

## Figures and Tables

**Table 1:**
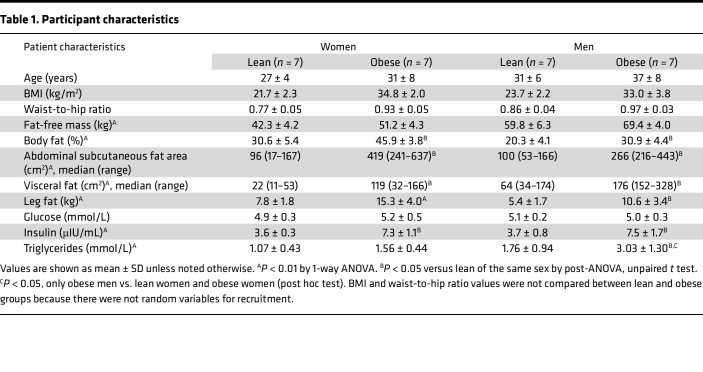
Participant characteristics

**Table 2:**
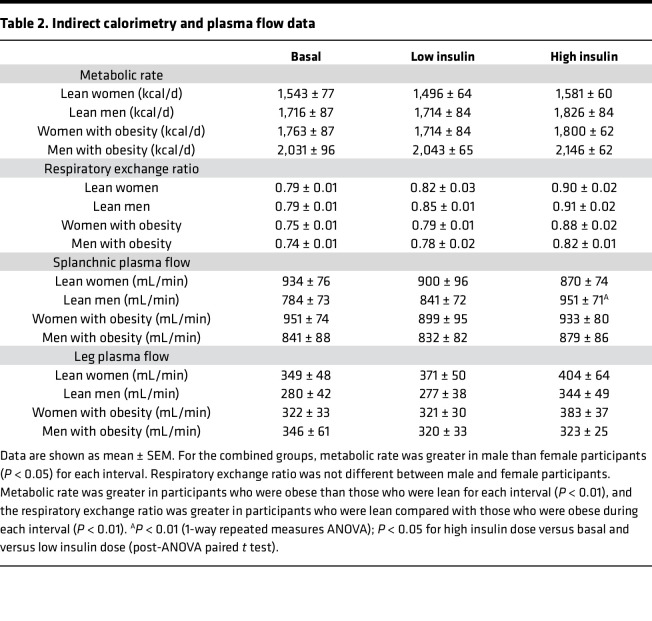
Indirect calorimetry and plasma flow data

**Table 3:**
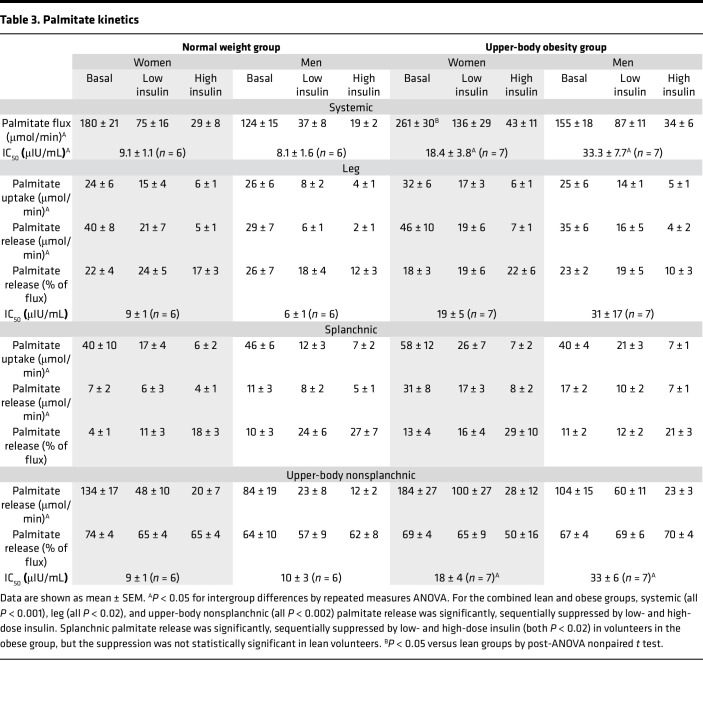
Palmitate kinetics

**Table 4:**
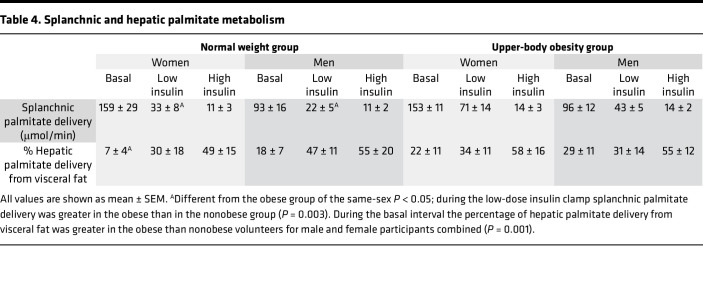
Splanchnic and hepatic palmitate metabolism

## Version 1 04/11/2024

In-Press Preview

## Version 2 05/08/2024

Electronic publication
