# Supplementary material for: Insulin regulation of regional lipolysis in upper-body obese and lean humans
Source: JCI Insight. 2024 Apr 11;9(9):e175629. doi: 10.1172/jci.insight.175629 (PMC11141918; doi:10.1172/jci.insight.175629)
Supplement: Supplemental data [file jciinsight-9-175629-s250.pdf]

Supplemental Figure 1

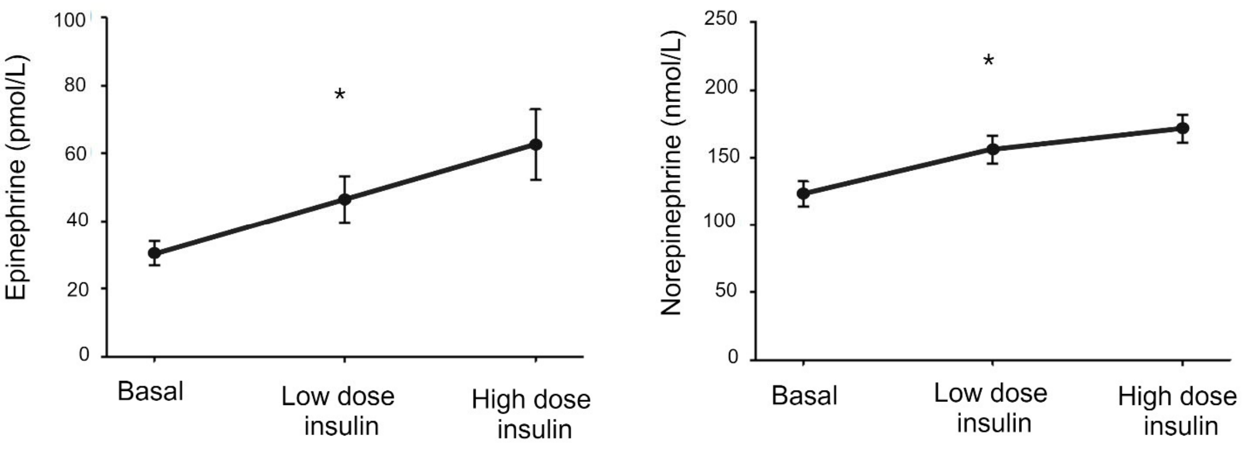

Supplemental Figure 1. Plasma epinephrine and norepinephrine concentrations during basal, low insulin dose infusion and high insulin dose infusion. *P<0.001, one-way repeated measures ANOVA. For plasma epinephrine concentrations - high dose insulin vs. low dose insulin, both P<0.05 (post hoc test). For plasma norepinephrine concentrations - high dose insulin vs. basal and low dose insulin vs. basal, all P<0.001 (post hoc test).

Supplemental Figure 2

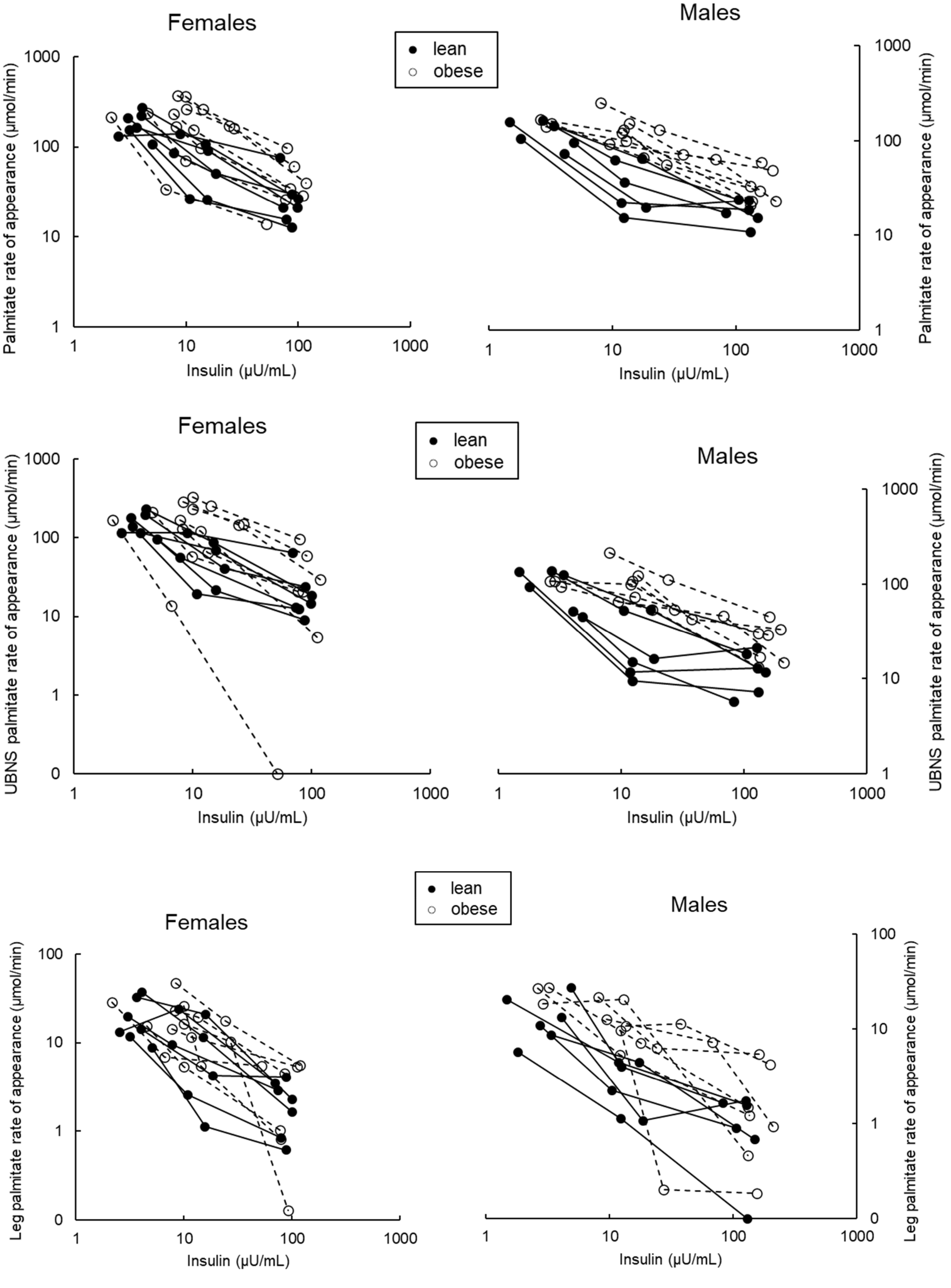

Supplemental Figure 2. Individual palmitate rates of appearance (log scale) are plotted vs. their individual plasma insulin concentrations (log scale) for males (right panels) and females (left panels) who were lean and who had the obese phenotype. The relationships between systemic palmitate rates of appearance and insulin concentrations are depicted in the upper panels, for upper body non-splanchnic (UBNS) of appearance in the middle panels and for leg palmitate rate of appearance in the lower panels.
